# Panniculus morbidus resection complicated by multiple gastrointestinal hernias: A case report

**DOI:** 10.1016/j.amsu.2022.104177

**Published:** 2022-07-16

**Authors:** Nolan M. Winicki, Isabella S. Florissi, Marcos J. Michelotti, Daniel P. Srikureja

**Affiliations:** a University of California Riverside, School of Medicine, Riverside, CA, USA; b Loma Linda University, Department of Surgery, Loma Linda, CA, USA; c Johns Hopkins University, School of Medicine, Baltimore, MD, USA

**Keywords:** Case report, Panniculus morbidus, Gastrointestinal hernia, Obesity

## Abstract

**Introduction:**

Prevalence of obesity and obesity-related complications are steadily rising in the United States. Panniculus morbidus is a rare end stage complication of abdominal obesity characterized by excess abdominal skin and subcutaneous tissue induced by severe lymphedema. The resulting pannus can limit a patient's mobility, impair activities of daily living including hygiene maintenance and subject the skin and soft tissue to intertrigo, cellulitis and chronic skin ulcerations.

**Case presentation:**

We present the case of a 39-year-old female with a BMI of 57 kg/m^2^ who presented for evaluation of primary umbilical and ventral hernias, as well as a large pannus causing significant abdominal and back pain. A massive panniculectomy with hernia repair was performed to correct the gastrointestinal herniation and panniculus.

**Clinical discussion:**

Panniculus morbidus is a debilitating complication of longstanding obesity. Massive panniculectomy is one of the only treatments available to restore functional status and facilitate future weight loss. Ventral and umbilical hernias commonly accompany panniculus morbidus and can pose a challenge to repair.

**Conclusion:**

This case demonstrates that both panniculus morbidus and multiple primary gastrointestinal hernias can be effectively managed with a panniculectomy and concomitant hernia repair with onlay mesh, all together safely improving patient ambulation, weight loss and quality of life.

## Highlights

• Panniculus morbidus incidence is increasing with the rise of morbid obesity.
• Gastrointestinal hernia repair is a challenging for patients with panniculus morbidus.
• Concomitant hernia repair and panniculectomy can be performed safely and effectively, enhancing weight loss and quality of life.

## 1 Introduction

Panniculus morbidus is a rare complication of abdominal wall obesity and is characterized by an excess of abdominal skin and subcutaneous tissue typically caused by severe lymphoedema [1]. The panniculus can become a nidus for chronic cellulitis, skin rashes, ulcers, fistulas, and ischemic panniculitis [2]. Additionally, obesity has been established as a risk factor for the development of primary and incisional ventral hernia, and specifically patients with panniculus have been shown to develop an infraumbilical abdominal hernia [3]. Due to a population-wide increase in morbid obesity and its associated complications, surgeons will more frequently need to repair complex hernias and should consider simultaneous panniculectomy in patients with panniculus morbidus complicated by gastrointestinal hernias. This case report has been reported in line with the SCARE Criteria [4].

## 2 Presentation of case

**Fig. 1:**
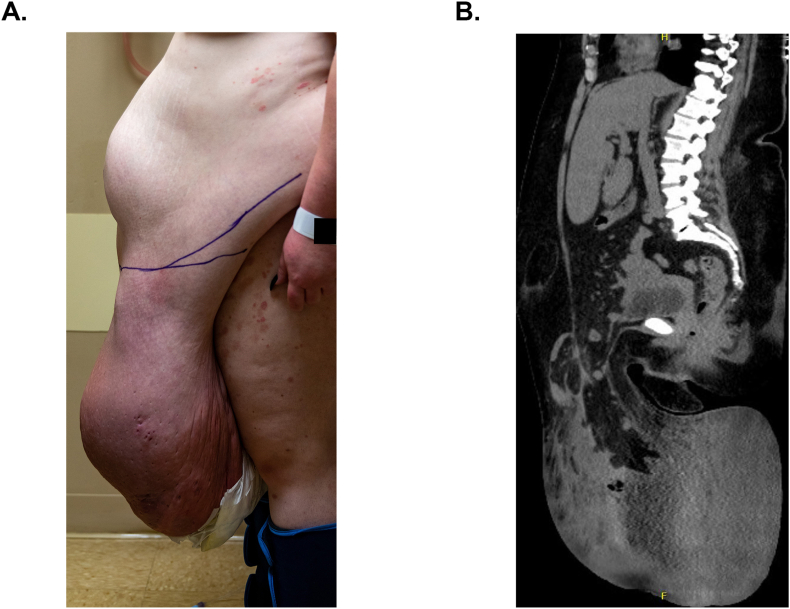
Preoperative imaging.

The patient is a 39-year-old female with a medical history of super morbid obesity (BMI 57 kg/m^2^), atrial fibrillation, hypertension and hyperlipidemia presenting for evaluation of primary umbilical and ventral hernias, as well as a large pannus causing significant abdominal and back pain. The umbilical hernia was noted to worsen over her 2 pregnancies, and the low midline hernia developed after cesarean section. The pannus was reported to have continually grown in size, despite weight loss efforts, and served as a nidus for infection and severely limited mobility (Fig. 1A). The patient denied any obstructive symptoms, but the hernias were not reducible on exam and appeared chronically incarcerated. A panniculectomy with hernia repair was scheduled to correct the gastrointestinal herniation and improve patient quality of life. Patient's pre-operative weight was 343 lbs. (155 kg).

Preoperative computed tomography (CT) scans displayed a ventral abdominal hernia comprised of peritoneal fat and nondilated bowel, measuring 3.6 cm in length, as well as a right lower quadrant ventral hernia comprised of mesenteric fat and bowel, measuring 4.5 cm in length (Fig. 1B). Additionally, fat and non-dilated bowel was herniated into the pannus, causing extensive inflammatory change, skin ulceration and lymphedema fluid collection along the full length (10.7 cm) of the pannus.

**Fig. 2:**
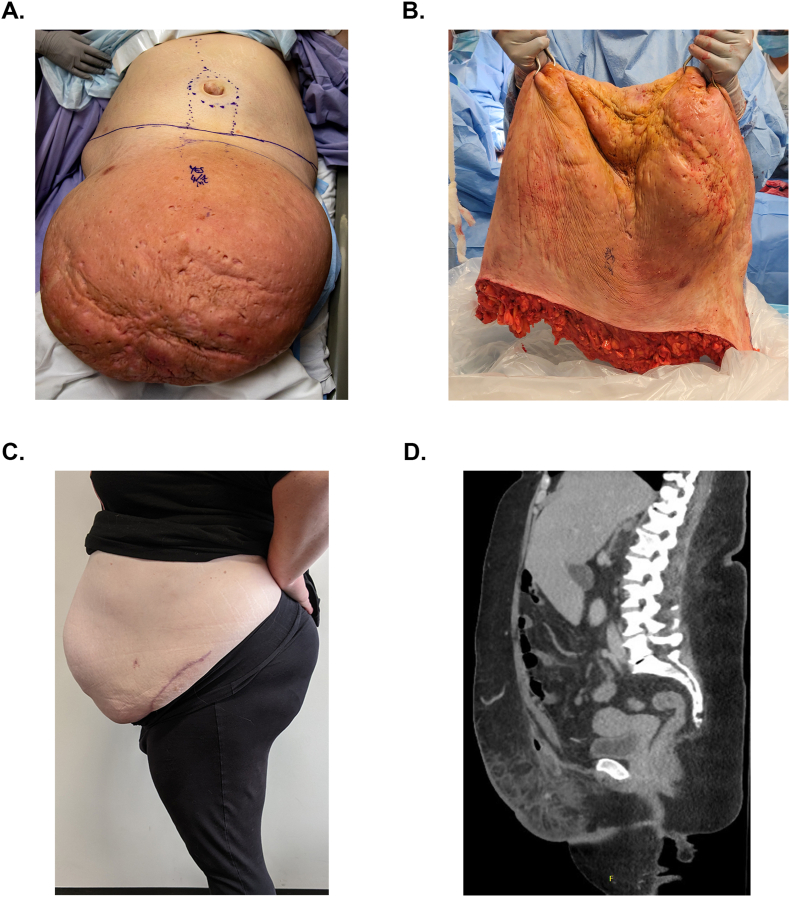
Operative approach, postoperative follow-up and imaging.

In the operating room (OR), the pannus was sterilized circumferentially and suspended to facilitate dissection. Surgical booms in the OR were maneuvered to sustain the weight of the pannus. Dissection was guided by curvilinear skin lines marked preoperatively that followed the suprapubic crease circumferentially around the pannus from one hip bone to the other (Fig. 2A). Significant edema surrounded the pannus, and numerous dilated veins in subcutaneous tissues were suture ligated to prevent hemorrhage. Due to the significant risks of fluid shift and hemorrhage, two attending surgeons worked simultaneously to reduce OR time. Dissection was carried circumferentially until the hernia sac was identified and the fascial defect was isolated. Operative blood loss was estimated at 1 L. The fascial defect was noted to be approximately 10 cm × 10 cm with both small and large bowel contained within the sac. The bowel was dissected from the sac and reduced into the abdomen. The defect was closed with interrupted polydioxanone (PDS) suture, and a 15 cm × 20 cm Synecor (GORE, W. L. Gore & Associates Inc., Newark, DE, USA) mesh was placed in an onlay fashion overlying the closure and was buttressed to the anterior fascia with running 2–0 PDS Stratafix (Ethicon, Johnson & Johnson, Cincinnati, OH, USA) sutures bilaterally. Skin and subcutaneous flaps were raised to allow for tension free closure, which was performed with 2–0 interrupted Vicryl (Ethicon, Johnson & Johnson, Cincinnati, OH, USA) deep dermal sutures and 4-0 Monocryl (Ethicon, Johnson & Johnson, Cincinnati, OH, USA) sutures running subcuticular. Case length was 5 hours and 18 minutes.

After excision and delivery, the pannus measured 62 cm × 45 cm x 8.5 cm and weighed 35 lbs. (15.8 kg) (Fig. 2B). Pathology report of the pannus showed diffusely scattered defects with tracts filled with firm tan-white friable purulent exudate and severely edematous subcutaneous tissue. Patient's postoperative weight was 295 lbs. (134 kg). Based on the differences in pre- and post-operative weight, we deduced that the patient lost over 13 lbs. (6 kg) of fluid during the case.

The patient recovered well and was discharged on postoperative day three, ambulating without difficulty, and tolerating a normal oral diet. Close follow-up care and weight loss recommendations were conducted. At this time, the patient is satisfied with her improvement in quality of life and has observed significant advancement in mobility and in her ability to participate in activities of daily living. At 13 months follow-up, the patient's hernias have not recurred. However, the patient has still struggled with weight loss, maintaining a weight of 290 lbs. with a BMI of 48 kg/m^2^ (Fig. 2C and D). Referrals for medically supervised weight loss, and nutrition were placed. The patient is currently attempting to lose weight independently and declined further options of bariatric surgery to meet BMI criteria (<40 kg/m^2^) for elective umbilical hernia repair.

## 3 Discussion

Panniculus morbidus is a rare condition in which a layer of abdominal fat and accompanying lymphedema overhangs the waistline, adversely impacting patient quality of life by limiting mobility, causing backpain, and serving as a nidus for recurrent infection [5]. Factors associated with morbid obesity, including obstruction of lymphatic channels and superficial veins, contribute to the development of panniculus morbidus [6]. Gravity-dependent accumulation of transudate occurs in dependent parts of the panniculus, stretching overlying skin and aggravating the lymphatic obstruction, promoting further growth of the pannus [5]. As the incidence of obesity is steadily increasing, so will the incidence of panniculus morbidus [7]. However, the diagnosis of panniculus morbidus is often missed until patient quality of life is severely diminished. As such, early diagnosis and treatment is essential to diminishing pathological severity and morbidity, and potentially mitigating pannus-related complications [8].

Previous case studies have documented the removal of panniculus morbidus either prior to or following bariatric surgery and have outlined potential complications of panniculectomy with conflicting results over the influence of simultaneous incisional hernia repair on post-operative complications [9,10]. However, this report outlines the safe and effective surgical repair of panniculus morbidus complicated by multiple primary gastrointestinal hernias resulting in no major postoperative complications. In these patients, panniculectomy can be performed to improve patient quality of life, reduce pain, improve mobility, facilitate weight loss, and aid in the reduction of hernias, which are often associated with the increased size of a pannus or worsening of lymphedema [8]. We show that concomitant ventral hernia repair is optimal during the panniculectomy, but still recommend to postpone elective abdominal hernia repair until patient BMI is under 40 kg/m^2^.

Intraoperative management of a pannus can be complicated by its large dimensions and weight. In this case, the weight of the pannus was supported by surgical booms, which may serve as a more accessible method for operative support. Previous literature has also recommended the use of holding forceps supported by a metal bar, Steinmann pins, or the help of a surgical assistant [6].

## 4 Conclusion

Existing literature regarding the treatment of abdominal panniculus morbidus is limited and does not fully explore techniques for the safe concomitant management of multiple gastrointestinal hernias. This case report demonstrates that both panniculus morbidus and gastrointestinal hernias can be effectively managed concomitantly with a panniculectomy and primary hernia repair with onlay mesh, thus improving patient quality of life, reducing pain, improving mobility, and facilitating future weight loss.

## Sources of funding

None.

## Ethical approval

The patient was informed, and we have acquired her consent for this publication.

## Consent

Written informed consent was obtained from the patient for publication of this case report and accompanying images. A copy of the written consent is available for review by the Editor-in-Chief of this journal on request.

## Author contribution

NW, IF, MM and DS: wrote the manuscript, DS and MM: surgical treatment for this patient, DS and MM: study design, NW, IF: data interpretation, DS and MM: patient care.

## Registration of research studies

Name of the registry:
Unique Identifying number or registration ID:
Hyperlink to your specific registration (must be publicly accessible and will be checked):

## Guarantor

Nolan M. Winicki, MS is the guarantor for this paper.

## Provenance and peer review

Not commissioned, externally peer-reviewed.

## Declaration of competing interest

None.

## References

[1] Felmerer G., Karcz W., Földi E., Tobbia D. (2012/09/01 2012). Integrated concept of treatment for reduction of morbidity after resection of panniculus morbidus associated with lymphoedema. Journal of Plastic Surgery and Hand Surgery.

[2] Petty P., Manson P.N., Black R., Romano J.J., Sitzman J., Vogel J. (1992). Panniculus morbidus. Ann. Plast. Surg..

[3] Sugerman H.J., Kellum J.M., Reines H.D., DeMaria E.J., Newsome H.H., Lowry J.W. (1996). Greater risk of incisional hernia with morbidly obese than steroid-dependent patients and low recurrence with prefascial polypropylene mesh. *Am J Surg*. Jan.

[4] Agha R.A., Franchi T., Sohrabi C., Mathew G., Kerwan A. (Dec 2020). The SCARE 2020 guideline: updating consensus surgical CAse REport (SCARE) guidelines. Int. J. Surg..

[5] Petty P., Manson P.N., Black R., Romano J.J., Sitzman J., Vogel J. (1992). Panniculus morbidus. Ann. Plast. Surg..

[6] Fadel M.G., Chatzikonstantinou M., Gilchrist C., Andrews B. (Apr 29 2017). Panniculus morbidus: obesity-related abdominal wall lymphoedema. BMJ Case Rep..

[7] MacDonald K.G. (2003). Overview of the epidemiology of obesity and the early history of procedures to remedy morbid obesity. Arch. Surg..

[8] Evans C., Debord J., Howe H., Marshall J.S., Rossi T., Owolabi M. (2014). Massive panniculectomy results in improved functional outcome. *Am J Surg*. Mar.

[9] Zemlyak A.Y., Colavita P.D., El Djouzi S. (2012/10/01/2012). Comparative study of wound complications: isolated panniculectomy versus panniculectomy combined with ventral hernia repair. J. Surg. Res..

[10] Warren J.A., Epps M., Debrux C. (2015/08/01 2015). Surgical site occurrences of simultaneous panniculectomy and incisional hernia repair. Am. Surg..

